# Detection of urinary podocytes by flow cytometry in idiopathic membranous nephropathy

**DOI:** 10.1038/s41598-020-73335-2

**Published:** 2020-10-01

**Authors:** Alberto Mella, Ilaria Deambrosis, Silvia Mingozzi, Loredana Colla, Manuel Burdese, Fulvia Giaretta, Stefania Bruno, Giovanni Camussi, Elena Boaglio, Caterina Dolla, Roberta Clari, Luigi Biancone

**Affiliations:** 1 Division of Nephrology Dialysis and Transplantation, Department of Medical Sciences, Città Della Salute e della Scienza Hospital, University of Turin, Corso Bramante, 88-10126 Turin, Italy; 2 Laboratory of Nephrology and Immunopathology, Città Della Salute e della Scienza Hospital, Turin, Italy; 3 Department of Medical Sciences, University of Turin, Turin, Italy

**Keywords:** Nephrology, Kidney, Kidney diseases

## Abstract

Idiopathic membranous nephropathy (iMN) is considered an immune-mediated disease where circulating autoantibodies against podocyte targets (mainly the PLA_2_R) cause the deposition of in-situ subepithelial immune-complexes. The consequent podocyte damage may cause cell detachment in urine (Podocyturia-PdoU). PdoU has been assessed in different kidney diseases, but limited data are available in iMN. In this study all patients with a diagnosis of iMN between 15/12/1999–16/07/2014 were tested for PLA_2_R antibodies (Ab anti-PLA_2_R, ELISA kit) and PdoU by flow cytometry with anti-podocalyxin antibody. A semi-quantitative PdoU score was defined according to the percentage of podocalyxin positive cells normalized to the total volume of sample and set relative to the urine creatinine measured in the supernatant. PdoU was positive in 17/27 patients (63%; 1+ score in 6/27—22.2%, 2+ in 4/27—14.8%, 3+ in 2/27—7.4%, 4+ in 5/27—18.5%). Only 2/7 patients with complete remission showed a positive PdoU (1+) while all six patients without remission have significant PdoU. PdoU+ was statistically correlated with the absence of remission and Ab anti-PLA_2_R + (p < 0.05) but PdoU, analysed as a continuous variable, showed a non-linear correlation with proteinuria or PLA_2_R antibody levels also in the cohort of patients with two available PdoU tests. In conclusion, PdoU could be detected in iMN and seems to be associated with commonly considered markers of disease activity (proteinuria and Ab anti-PLA_2_R) with a non-linear correlation. Despite data should be confirmed in large and prospective cohorts, according to the podocyte depletion hypothesis PdoU may represent an early marker of immunological activation with potential prognostic utility.

## Introduction

Membranous nephropathy (MN) is one of the most important cause of nephrotic syndrome in adults, with an incidence of about 1.7 cases per 100,000 inhabitants/year in the Caucasian population^1^. According to previous studies in animal models and humans^2,3^ MN may be considered as an immune-mediated disease, where circulating auto-antibodies determined the formation of subepithelial immune deposits followed by a widespread thickening of the glomerular capillary wall^4^. To date, research is focusing on the etiopathogenesis to identificate reliable biomarkers for monitoring disease progression and therapeutic response^5^.

In the majority of previously considered idiopathic MN (iMN, accounting 75–80% of all cases) circulating and implanted antibodies againt a constitutive podocyte receptor (Phospolipase A_2_ receptor; PLA_2_R) could be detected, and their levels correlate to disease activity, recovery and relapse^6,7^. Several experimental models suggest a crucial role for podocyte damage in determining a clinically relevant disease^8,9^, especially in case of podocyte detachment, a phenomenon called podocyturia (PdoU).

PdoU has been assessed in a wide range of kidney diseases, from Fabry to pre-eclampsia^10–12^ with different methods (immunohistochemistry/immunofluorescence^10,11^, flow cytometry^12^, polymerase chain reaction^13^). Limited data are available in MN and none of them, at the best of our knowledge, have associated PdoU to anti-PLA_2_R antibodies (Ab anti-PLA_2_R).

In this study we focused our attention on PdoU (analysed by flow cytometry) in patients with iMN correlating our findings to clinical outcome and Ab anti-PLA_2_R.

## Results

### Clinical characteristics of studied population

Baseline clinical characteristics of our population are summarized in Table 1. Briefly, of the twenty-nine patients with iMN diagnosed between 15/12/1999 and 16/07/2014 at our Nephrology Unit, 13 were males and 16 females with a mean age at diagnosis of 57.7 ± 12.87 years. At the time of the diagnosis most patients had a slightly reduced renal function (serum creatinine—sCr—1.1 ± 0.5 mg/dl, estimated glomerular filtration rate—eGFR—86.3 ± 33.2 ml/min/1.73m^2^) and a nephrotic-range proteinuria (PTU) (6.6 ± 4.4 g/24 h). Among the 25/29 (86%) patients with a PTU ≥ 3 g/24 h, fourteen out of 25 (56%) presented a selective glomerular proteinuria, and 12/25 (48%) a complete or incomplete tubular proteinuria.

**Table 1:** Clinical characteristics of studied population at diagnosis.

Charachteristics	Studied population (n = 29)
Sex (ratio M/F)	13/16 (0.8/1)
Age, years	57.7 ± 12.87
Serum creatinine, mg/dl	1.1 ± 0.5
Estimated glomerular filtration rate, ml/min/1.73m^2a^	86.3 ± 33.2
Proteinuria, g/24 h	6.6 ± 4.4
Follow-up, years	7.26 ± 3.97

All data are expressed as mean ± DS.

^a^Estimated with CKD-EPI formula.

All patients were managed with an immunosuppressive (24/29, 82.8%) or conservative treatment (Angiotensin converting enzyme inhibitors and/or Angiotensin type II receptor blockers) (5/29, 17.2%) at a mean time of 50.2 ± 86.9 days after diagnosis. Two out of 5 patients (40%) who were initially managed with a conservative approach received an immunosuppressive treatment during the f/up. According to KDIGO Guidelines^14^ most of the patients were treated with Cyclical corticosteroid/Alkylating-agent therapy for 6 months (14/24, 58.3%) or Cyclosporine A (8/24, 33.3%). Rituximab, Adrenocorticotropic hormone, Mycophenolate Mofetil, or Calcineurine inhibitors were adopted as second-line or rescue therapies in subjects (14/24, 58.3%) who failed to achieve remission after first-line treatment.

The mean f/up was 7.26 ± 3.97 years. At last evaluation, 24/29 patients (82.8%) were in complete (10/29, 34.5%) or partial (14/29, 48.3%) remission. Only in 5/29 patients (17.2%) a nephrotic-range proteinuria persisted despite different therapies.

During the f/up 11/29 (37.9%) patients experienced, after complete or partial remission, a disease recurrence up to a maximum of three episodes/patient. A history of recurrence was significantly correlated with the absence of remission at the end of f/up (OR 2.5 95% CI 1.17–5.3, p < 0.05); none of the other clinical variables (sex, age, sCr/eGFR/PTU at diagnosis, therapy) was correlated with disease remission.

### *Evaluation of podocyturia and Ab anti-PLA*_*2*_*R*

Patients were tested for PLA_2_R antibodies and PdoU at a mean time after diagnosis of 5.24 ± 3.35 years; two subjects were escluded from the analysis due to a persistent leukocyturia which may interfere with PdoU analysis.

Eleven out of 29 (38%) patients were Ab anti-PLA_2_R antibody positive, with a mean antibody level of 106.5 ± 81 RU/ml. PdoU was positive in 17/27 patients (63%), with a semiquantitative 1+ score in 6/27 (22.2%), 2+ in 4/27 (14.8%), 3+ in 2/27 (7.4%) and 4+ in 5/27 (18.5%).

Stratifying PdoU according to disease remission at the time of the test, only 2/7 patients with complete remission showed a positive PdoU (in both cases at the minimum 1+ score) while all six patients without remission have positive PdoU. PdoU expressed as a discrete variable (pos/neg) was also statistically correlated with the absence of remission and a positive test for Ab anti-PLA_2_R (p < 0.05 for both analysis).

**Figure 1:**
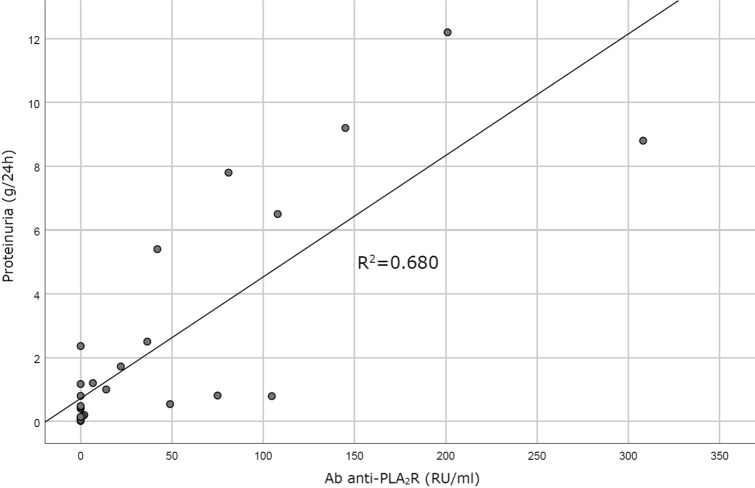
Correlation between Ab anti-PLA_2_R and proteinuria. Ab anti-PLA_2_R and proteinuria shows a linear correlation (R^2^ = 0.6891, p < 0.005).

**Figure 2:**
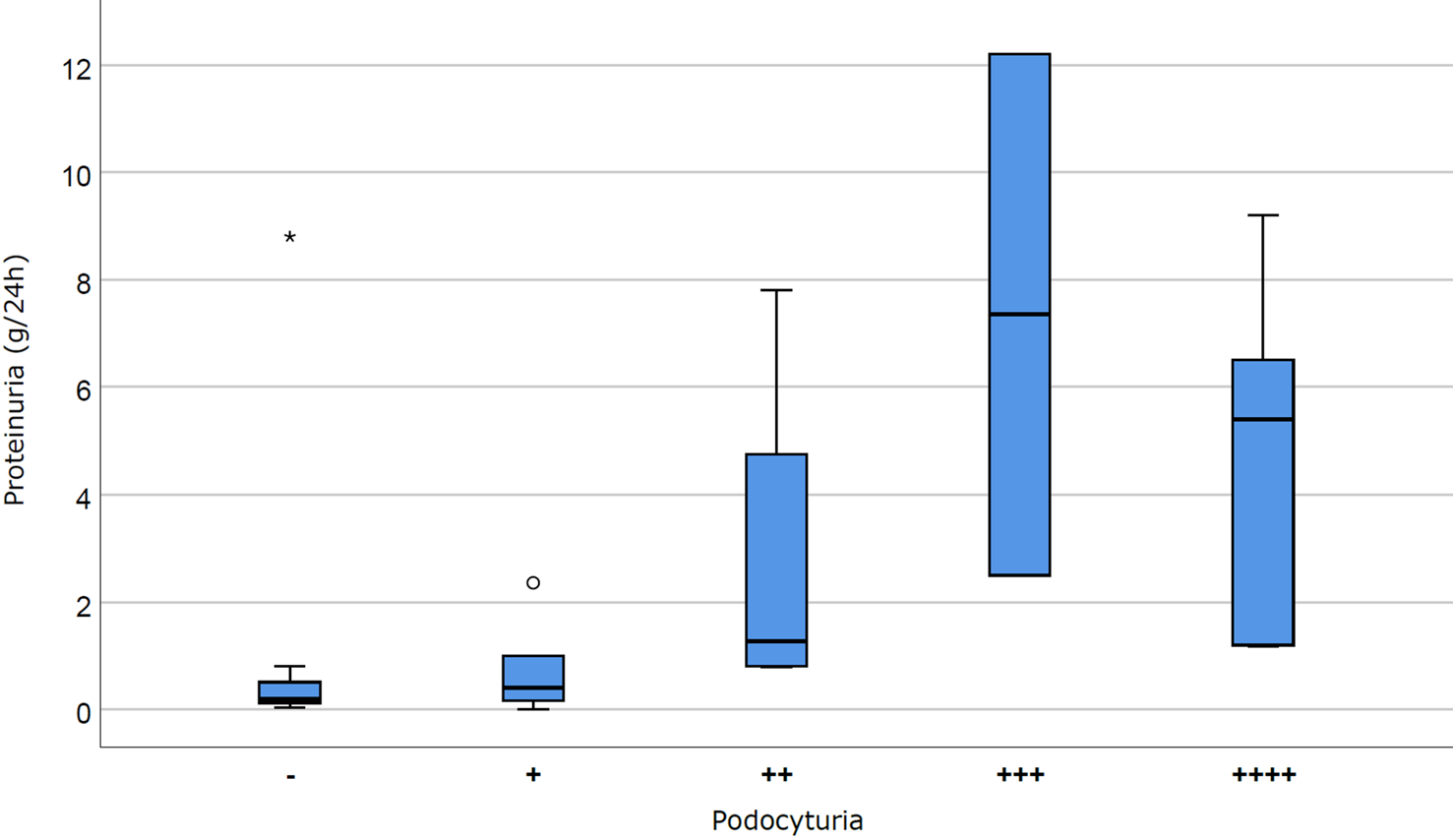
Correlation between podocyturia (*semiquantitative* *score*) and proteinuria values. Podocytura and proteinuria seem to not be linearly correlated (p = NS).

**Figure 3:**
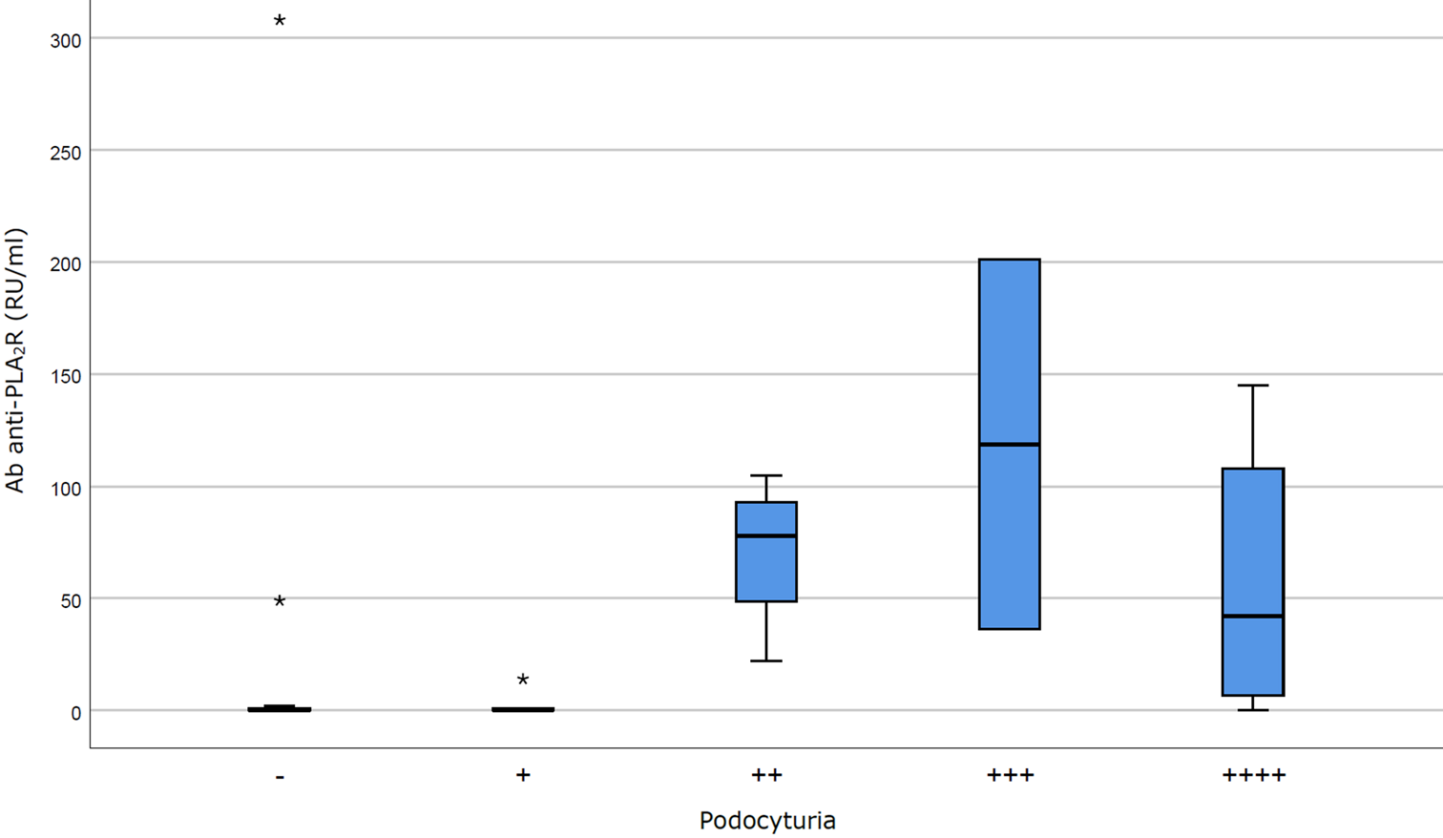
Correlation between podocyturia (*semiquantitative* *score*) and Ab anti-PLA_2_R. As for proteinuria, podocytura and Ab anti-PLA_2_R seem to not be linearly correlated (p = NS).

Despite a statistically significant direct correlation was shown between Ab anti-PLA_2_R levels and PTU (p < 0.05; Fig. 1), the analysis of PdoU as a continuous variable (according to both continuous values and semiquantitative score) showed no significant correlation between PdoU and disease remission, PTU (Fig. 2; Fig. S1 in Supplementary Material) or Ab anti-PLA_2_R level (Fig. 3; Fig. S2 in Supplementary Material). The possibility of a non-linear correlation has been exemplified in Fig. 2, where 15/17 PdoU positive patients had a proteinuria ≥ 0.5 g/24 h, but PdoU did not seem to be directly correlated to proteinuria values.

The similar tendency was observed in the cohort of patients were a previous PdoU test was available during the f/up, and where increase of PTU is contemporary associated to PdoU decrease/increase (Table 2).

**Table 2:** Clinical characteristics and podocyturia score in the cohort of patients with two podocyturia determinations.

	sCr (mg/dl)	PTU (g/24 h)	PdoU first score^a^	sCr (mg/dl)	PTU (g/24 h)	PdoU second score^a^
Patient 1	2.40	1.4	Negative	3.86	9.2	Positive (4+)
Patient 2	2.40	2.4	Positive (3+)	2.4	5.4	Positive (4+)
Patient 3	1.93	10.7	Positive (4+)	2.22	7.8	Positive (2+)
Patient 4	1.00	1.1	Negative	2.24	8.8	Positive (1+)
Patient 5	2.30	7	Positive (4+)	2.51	12.2	Positive (3+)
Patient 6	0.73	6	Positive (2+)	1.16	0.16	Positive (1+)
Patient 7	1.00	4.4	Negative	1.23	0.48	Negative

*sCr* serum creatinine, *PTU* proteinuria, *PdoU* podocyturia.

^a^Median time between PdoU determinations 20.5 months (min 19–max 22).

No significant correlations were also found between PdoU and different treatments, despite all the 4 patients treated with Rituximab during the f/up (all in partial remission at the time of the test) were negative for PdoU.

## Discussion

iMN is now defined as an immune-mediated disease where circulating auto-antibodies against podocyte targets (mainly the PLA_2_R)^6^ cause the deposition of in situ sub-epithelial immune-complexes^15^. Ab anti-PLA_2_R levels during the f/up show a strong correlation with disease activity^7,16^, and the availability of commercial kits with high sensitivity/specificity for antibody detection is now changing the management of this condition^17^. On the other hand, although some clinical variables at presentation have been associated with a negative outcome (male sex, old age, nephrotic-range proteinuria/reduced eGFR), in many cases it is still impossible to recognize what will be the long-term outcome for a patient with iMN^18^.

Despite some limits (low numerosity, retrospective design, absence of serum at diagnosis) also in our experience no paramether or therapy is associated with clinical outcome; on the contrary Ab anti-PLA_2_R levels have a significant direct correlation with proteinuria values. At the same time, a statistically significant association between absence of remission and positive podocyturia (PdoU) was observed, without linear correlation between proteinuria and PdoU values.

PdoU showed a direct association with disease activity in a wide range of glomerulopathies^10–12^, and all different PdoU tests recognize the detached podocytes on the basis on a specific protein, primarily podocalyxin.

Podocalyxin is a CD34-related, extensively O-glycosylated and sialylated type-1 transmembrane protein, normally expressed in podocytes, haematopoietic progenitors, endothelial cells and a subset of neurons^19^. In the kidney, podocalyxin is predominantly located at the apical surface of podocytes^19,20^ and for these carachteristics as been widely adopted for PdoU analysis.

Some Authors have investigated the role of PdoU in patients with glomerulonephritis also including MN patients but in small cohorts often obtaining conflicting or inconclusive results. In the first experience by Hara et al.^21^ (PdoU analyzed in immunofluorence with anti-podocalyxin antibody) none of the 3 pediatric patients with MN have detectable urinary podocytes; also in a second case series of the same research group the 10 patients with MN showed a very low PdoU compared to patients with focal segmental glomerulonephritis^22^. No difference in PdoU (intended as mRNA expressions of three different podocyte proteins–nephrin, podocin and synaptopodin) between control and 5 patients with MN was reported by Szeto et al.^23^. In the most consistent experience available in Literature, Wickman et al.^13^ evaluated the degree of urinary mRNA levels of nephrin, podocin, TGF-b1, and aquaporin-2 in 649 patients with several gomerulopathies, including 15 patients with MN. They found a significant relationship between proteinuria and podocyturia detachment mainly in advanced diabetic nephropathy, in progressors and in active lupus nephritis or IgA nephropathy, but a low correspondance in MN. However, our results were similar to the observation by Achenbach et al. who documented an increase in podocalyxin-positive urinary cells in 11 MN patients with active disease vs 12 patients on partial/complete remission^24^, despite with their adopted approach (immunocytochemistry after overnight culture) Authors may not definitely discriminate between excreted podocytes or parietal epithelial cells.

**Figure 4:**
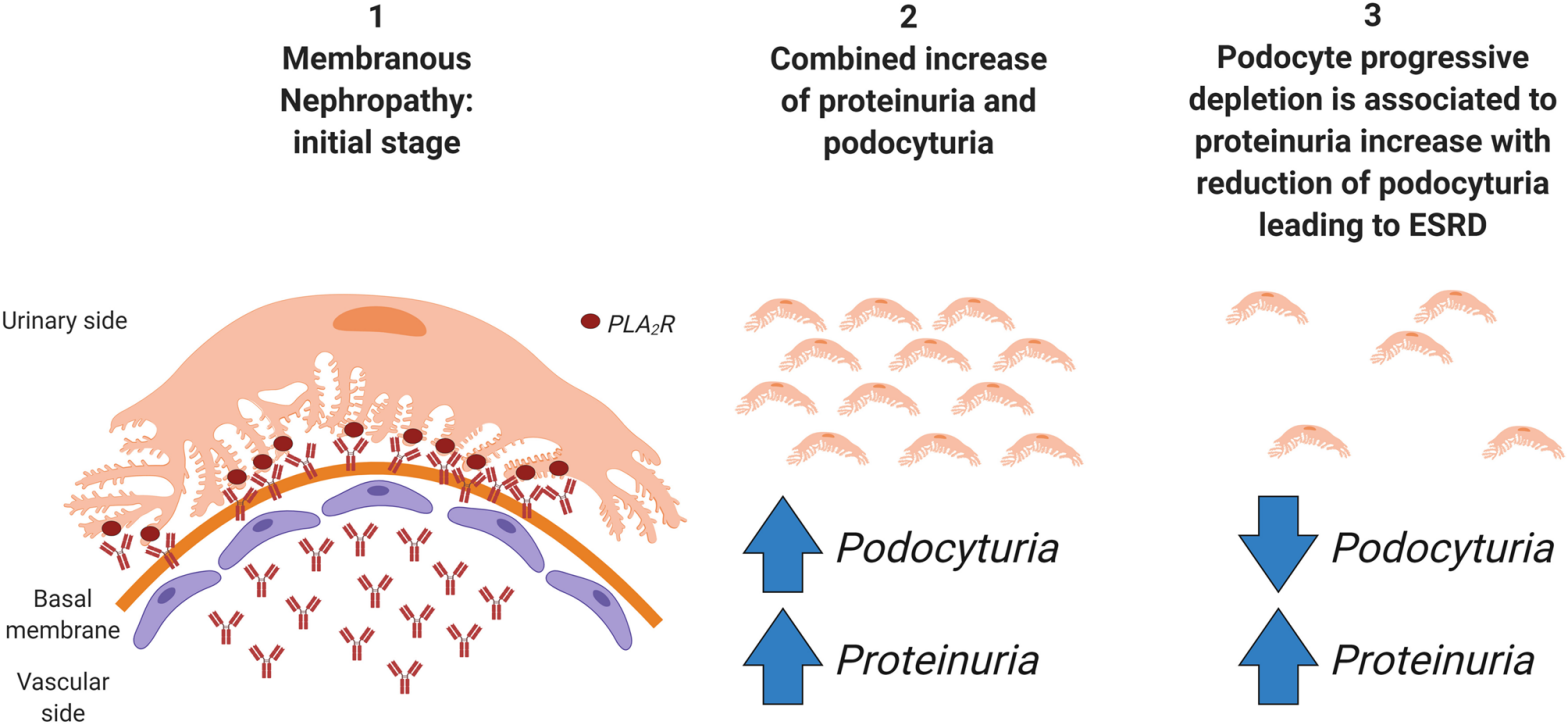
Graphical schematization of idiopathic membranous nephropathy in the context of podocyte depletion hypothesis. (1) In the initial stage, Ab anti-PLA_2_R caused immunocomplexes-deposition and podocyte damage after interaction with their podocyte target; (2) after the initial damage, podocytura and proteinuria both increase; (3) in case of persisting immunological damage, the reduction of podocyte mass progressively leads to end-stage renal disease (ESRD) with podocyturia decrease and, per contrast, further increase in proteinuria. This figure is created with BioRender.com.

The evidence of a non-linear correlation between proteinuria and podocyte excretion has also been previously considered^13,25^: Trimarchi^25^ in example suggests that the connection between PdoU and proteinuria depends on the stage at which the process is approached. According to podocyte depletion hypothesis, progression to end-stage renal disease is driven by a progressive podocyte loss^26,27^. In this theory, proteinuria is a non-specific marker of glomerular injury and would increase in combination of PdoU if effective therapies are unable to reduce the rate of podocyte detachment. Once the mass of podocytes is low, PdoU would decrease in contrast to a climb in proteinuria and a parallel decline in kidney function^11^. On the contrary, podocyte detachment in the initial stages is not only related to proteinuria, potentially providing some useful additional information about immunological disease activity, response to treatment and prognosis^13^. A graphical scheme of iMN in the context of podocyte depletion hypothesis is summarized in Fig. 4. These observations are confirmed in our cohort of patients where two PdoU determinations were available, and in which an increase in PTU was associated of both PdoU increase (suggesting an immunological activation and persistence of podocyte “functional” mass) or reduction (glomerular sclerosis without immunological involvement?).

In conclusion, considering the lack of reliable biomarkers for evaluating immunological activitation, PdoU ought to be a field to explore and to be standardized and validated for routine assessment of iMN patients. Our study has several limitations (sample size, clinical and PdoU evaluation during the f/up, absence of control group). Regarding our approach for PdoU detection, the cytofluorimetric evaluation is performed on “fresh” morning urine avoiding overnight culture (a condition that may cause dedifferentiation/selction of unspecific cell subtypes as suggested in Achenbach et al.^24^), and also adjusted to a population of immortalized podocytes in order to reduce the impact of possible interference by other cell fractions (i.e. parietal epithelial cells).

Despite these limits, our experience represents one of the larger cohort investigating the role of PdoU in iMN, and the first analysis who demonstrate a correlation between PdoU and Ab anti-PLA_2_R, paving the way to larger (ideally prospective) cohort studies.

## Methods

### Study design and samples collection

The study included all patients with a diagnosis of iMN in the period between 15/12/1999 and 16/07/2014. The diagnosis of the disease was performed by renal biopsy or, in cases where biopsy was considered contraindicated due to patient conditions (i.e. kidney malformations or excessive bleeding risk), by a positive PLA_2_R antibody assay (immunofluorescence and/or ELISA). The f/up ended at 31/01/2018. Patients lost to f/up (including deaths), with a documented secondary MN or without a minimum of one control/year were excluded.

All clinical data were collected from the discharge letters and the reports of the outpatient visits. Evidence of remission (complete, partial or absence) was defined according to KDIGO 2012 Guidelines^14^. Sera and urine samples were collected on the same day during f/up.

Ab anti-PLA_2_R were determined by an enzyme-linked immunosorbent assay kit (Euroimmun, Medizinische Labordiagnostika, AG).

The study has been approved by our internal Ethical Committee and was conducted in accordance with good international clinical practice guidelines. Informed consent about study protocol was obtained from all patients.

### Qualitatitative and quantitative analysis of urinary podocytes (PdoU)

Urine samples were immediately prepared after collection for evaluation following the methods described by Perez-Hernandez et al.^28^ with modification. Briefly, 50 ml of the first morning urine was transferred in two sterile tubes and centrifuged for 6 min at 1600 rpm (centrifuge: Heraeus Megafuge 1.0). Sediments were washed with sterile phosphate-buffered saline, centifuged for 5 min at 1500 rpm twice, and then resuspended in 1 ml of sterile phosphate-buffered saline with 20% of cell culture medium and 5% of fetal bovine serum. For podocytes quantification 100 µl from the first tube were incubated with 10 µl Mouse Anti-Human Podocalyxin Phycoerythrin-conjugated Monoclonal Antibody (R&D Systems, Minneapolis, MN, USA) for 30 min at 4 °C in the dark; the same volume from the second tube was incubated with 15 μL mouse IgG_2_A Allophycocyanin-conjugated isotype control antibody (R&D Systems, Minneapolis, MN, USA).

In some patients with significant PdoU, after cell fixation and permeabilization (Fix and Perm, Caltag Laboratories, Burlingame, CA, USA), a confirmation with an intra-cytoplasmic staining with 5 µl Rabbit-Anti-human-Podocin Alexa Fluor 488 Conjugated antibody (Bioss Inc, Woburn, MA, USA) was obtained (data not shown).

**Figure 5:**
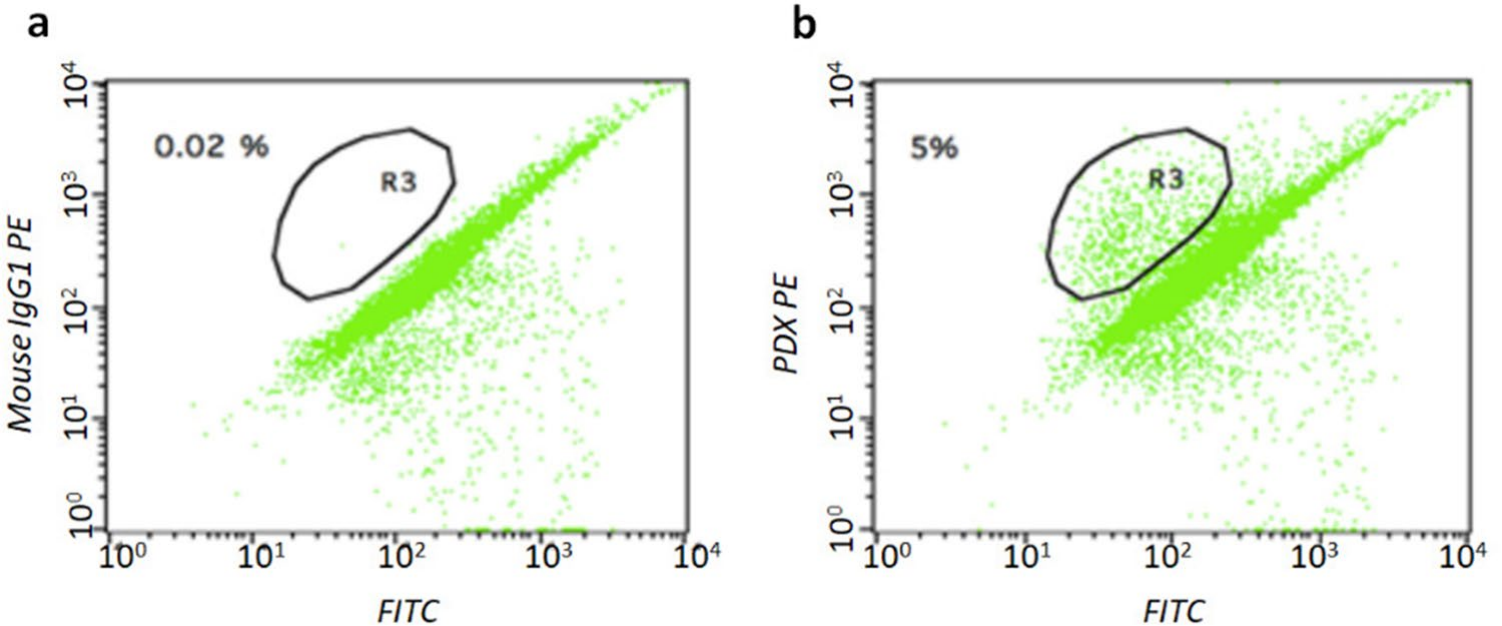
PdoU analysis (*flow* *cytometry*). (**a**) isotype negative control (**b**) podocalyxin positive cells (PDX PE) on the R3-gate.

For the assay, 50,000 cells were analysed by flow cytometer (BD FACSCalibur, Becton Dickinson). The whole procedure was perfomed by a single operator who was unaware of the patient’s condition to minimize intra-operator variability. A population of immortalized podocytes, obtained as previously described^29,30^, was used to identify the range of physical parameters, dimensions and internal organization characteristic of these cells, and to identify a region (R3 gate). Therefore, the positive podocalyxin elements were selected in this gate, which remained constant in all analyses (Fig. 5). The number of podocalyxin-positive cells was normalized to the total volume of the urine sample and set relative to the urine creatinine measured in the supernatant; a semi-quantitative score was than defined according to the number of podocalyxin positive cells per unit of creatinine (PdoU score 1+: 0–9 podocalyxin positive cells per mg urinary creatinine; PdoU score 2+: 10–19 podocalyxin positive cells per mg urinary creatinine; PdoU score 3+: 20–29 podocalyxin positive cells per mg urinary creatinine; PdoU 4+  ≥ 30 podocalyxin positive cells per mg urinary creatinine).

### Statistical analysis

Statistical analysis was performed with Spss (IBM SPSS Statistics, verse 22.0.0). The continuous variables were analyzed with the Kolmogorov–Smirnov test for the verification of the normal distribution; in the case of non-normal distributions, these variables were described with the median (min–max) and the difference between groups was verified with non-parametric tests (Mann–Whitney, Wilcoxon or Kruskal–Wallis). In the case, instead, of normal distributions, they have been described with mean ± standard deviation (SD) and the difference between groups has been verified with t test for independent or coupled samples. The categorical variables were described with fractions and the difference in prevalence was verified with Chi-Square (Pearson) or, if necessary, with the exact Fisher test; when appropriate, the odd ratio was calculated as a Relative Risk estimate. Box and dispersion charts were used. The level of significance (α) was set at 0.05.

### Ethical statement

The study protocol was approved by AOU Città della Salute e della Scienza Internal Ethical Comitee. The study was conducted in accordance with Declaration of Helsinki.

## Supplementary information

Supplementary Information 1

## Data Availability

All data and datasets used and/or analysed during the current study are available from the corresponding author on reasonable request.

## References

[1] Glassock RJ (2010). The pathogenesis of idiopathic membranous nephropathy: A 50-year Odyssey. Am. J. Kidney Dis..

[2] Jefferson JA, Pippin JW, Shankland SJ (2010). Experimental models of membranous nephropathy. Drug Discov. Today Dis. Model..

[3] Farquhar MG (1994). gp330 and RAP: The Heymann nephritis antigenic complex. Ann. N. Y. Acad. Sci..

[4] Cattran DC (2001). Idiopathic membranous glomerulonephritis. Kidney Int..

[5] McGrogan A, Franssen CFM, De Vries CS (2011). The incidence of primary glomerulonephritis worldwide: A systematic review of the literature. Nephrol. Dial. Transplant..

[6] Beck LH (2009). M-type phospholipase A2 receptor as target antigen in idiopathic membranous nephropathy. N. Engl. J. Med..

[7] Hoxha E (2014). Phospholipase A2 receptor autoantibodies and clinical outcome in patients with primary membranous nephropathy. J. Am. Soc. Nephrol..

[8] Nangaku M, Shankland SJ, Couser WG (2005). Cellular response to injury in membranous nephropathy. J. Am. Soc. Nephrol..

[9] Liu W (2019). Immunological pathogenesis of membranous nephropathy: Focus on PLA2R1 and its role. Front. Immunol..

[10] Jim B (2012). Podocyturia as a diagnostic marker for preeclampsia amongst high-risk pregnant patients. J. Pregnancy.

[11] Trimarchi H (2016). Podocyturia is significantly elevated in untreated vs treated Fabry adult patients. J. Nephrol..

[12] Perez-Hernandez J (2016). Urinary dedifferentiated podocytes as a non-invasive biomarker of lupus nephritis. Nephrol. Dial. Transplant..

[13] Wickman L (2013). Urine podocyte mRNAs, proteinuria, and progression in human glomerular diseases. J. Am. Soc. Nephrol..

[14] Kidney Disease Improving Global Outcomes (2012). KDIGO Clinical practice guideline for glomerulonephritis. Kidnet Int. Suppl..

[15] Ronco P, Debiec H (2015). Pathophysiological advances in membranous nephropathy: Time for a shift in patient’s care. Lancet.

[16] Radice A (2016). Clinical usefulness of autoantibodies to M-type phospholipase A2 receptor (PLA2R) for monitoring disease activity in idiopathic membranous nephropathy (IMN). Autoimmun. Rev..

[17] De Vriese AS, Glassock RJ, Nath KA, Sethi S, Fervenza FC (2017). A proposal for a serology-based approach to membranous nephropathy. J. Am. Soc. Nephrol..

[18] Ponticelli C, Passerini P (2010). Can prognostic factors assist therapeutic decisions in idiopathic membranous nephropathy?. J. Nephrol..

[19] Nielsen JS, McNagny KM (2009). The role of podocalyxin in health and disease. J. Am. Soc. Nephrol..

[20] Hara M (2005). Apical cell membranes are shed into urine from injured podocytes: A novel phenomenon of podocyte injury. J. Am. Soc. Nephrol..

[21] Hara M (1998). Urinary excretion of podocytes reflects disease activity in children with glomerulonephritis. Am. J. Nephrol..

[22] Hara M, Yanagihara T, Kihara I (2001). Urinary podocytes in primary focal segmental glomerulosclerosis. Nephron.

[23] Szeto CC (2005). Messenger RNA expression of glomerular podocyte markers in the urinary sediment of acquired proteinuric diseases. Clin. Chim. Acta.

[24] Achenbach J (2008). Parietal epithelia cells in the urine as a marker of disease activity in glomerular diseases. Nephrol. Dial. Transplant..

[25] Trimarchi H (2017). Podocyturia: Potential applications and current limitations. World J. Nephrol..

[26] Kriz W, Gretz N, Lemley KV (1998). Progression of glomerular diseases: Is the podocyte the culprit?. Kidney Int..

[27] Wiggins RC (2007). The spectrum of podocytopathies: A unifying view of glomerular diseases. Kidney Int..

[28] Perez-Hernandez J (2018). Urinary podocyte-associated molecules and albuminuria in hypertension. J. Hypertens..

[29] Conaldi PG (1997). Distinct pathogenic effects of group B coxsackieviruses on human glomerular and tubular kidney cells. J. Virol..

[30] Conaldi PG (1998). HIV-1 kills renal tubular epithelial cells in vitro by triggering an apoptotic pathway involving caspase activation and Fas upregulation. J. Clin. Invest..

