# Author Correction: Detection of urinary podocytes by flow cytometry in idiopathic membranous nephropathy

**DOI:** 10.1038/s41598-020-79997-2

**Published:** 2020-12-18

**Authors:** Alberto Mella, Ilaria Deambrosis, Silvia Mingozzi, Loredana Colla, Manuel Burdese, Fulvia Giaretta, Stefania Bruno, Giovanni Camussi, Elena Boaglio, Caterina Dolla, Roberta Clari, Luigi Biancone

**Affiliations:** 1 Division of Nephrology Dialysis and Transplantation, Department of Medical Sciences, Città Della Salute E Della Scienza Hospital, University of Turin, Corso Bramante, 88-10126 Turin, Italy; 2 Laboratory of Nephrology and Immunopathology, Città Della Salute E Della Scienza Hospital, Turin, Italy; 3 Department of Medical Sciences, University of Turin, Turin, Italy

Correction to: *Scientific Reports* 10.1038/s41598-020-73335-2, published online 01 October 2020

The Supplementary file that accompanies this Article contains a typographical error in the spelling of the author.

“Ilaria De Ambrosis”

should read:

“Ilaria Deambrosis”

