# Supplementary material for: Detection of urinary podocytes by flow cytometry in idiopathic membranous nephropathy
Source: Sci Rep. 2020 Oct 1;10:16362. doi: 10.1038/s41598-020-73335-2 (PMC7530666; doi:10.1038/s41598-020-73335-2)
Supplement: Supplementary file 1 — Supplementary Information 1 [file 41598_2020_73335_MOESM1_ESM.docx]

**Supplementary information to:**

**Detection of urinary podocytes by flow cytometry in idiopathic membranous nephropathy**

**Alberto Mella^1^, Ilaria De Ambrosis^2^, Silvia Mingozzi^1^, Loredana Colla^1^, Manuel Burdese^1^, Fulvia Giaretta^2^, Stefania Bruno^2,3^, Giovanni Camussi^3^, Elena Boaglio^1^, Caterina Dolla^1^, Roberta Clari^4^ and Luigi Biancone^1*^**

^1^ Division of Nephrology Dialysis and Transplantation, Città della Salute e della Scienza Hospital and Department of Medical Sciences, University of Turin, Turin, Italy

^2^ Laboratory of Nephrology and Immunopathology, Città della Salute e della Scienza Hospital, Turin, Italy

^3^ Department of Medical Sciences, University of Turin, Turin, Italy

^4^ Department of Nephrology and Dialysis, Ospedale Maggiore di Chieri, Chieri, Italy

Content:

Page 2: Correlation between podocyturia (*continuous values*) and proteinuria

Page 3: Correlation between podocyturia (*continuous values*) and Ab anti-PLA_2_R

**Figure S1**. **Correlation between podocyturia (*continuous values*) and proteinuria.** a) Podocytura and proteinuria seem to not be linearly correlated (R^2^=0.256); b) Schematization of linear trend

**a)**


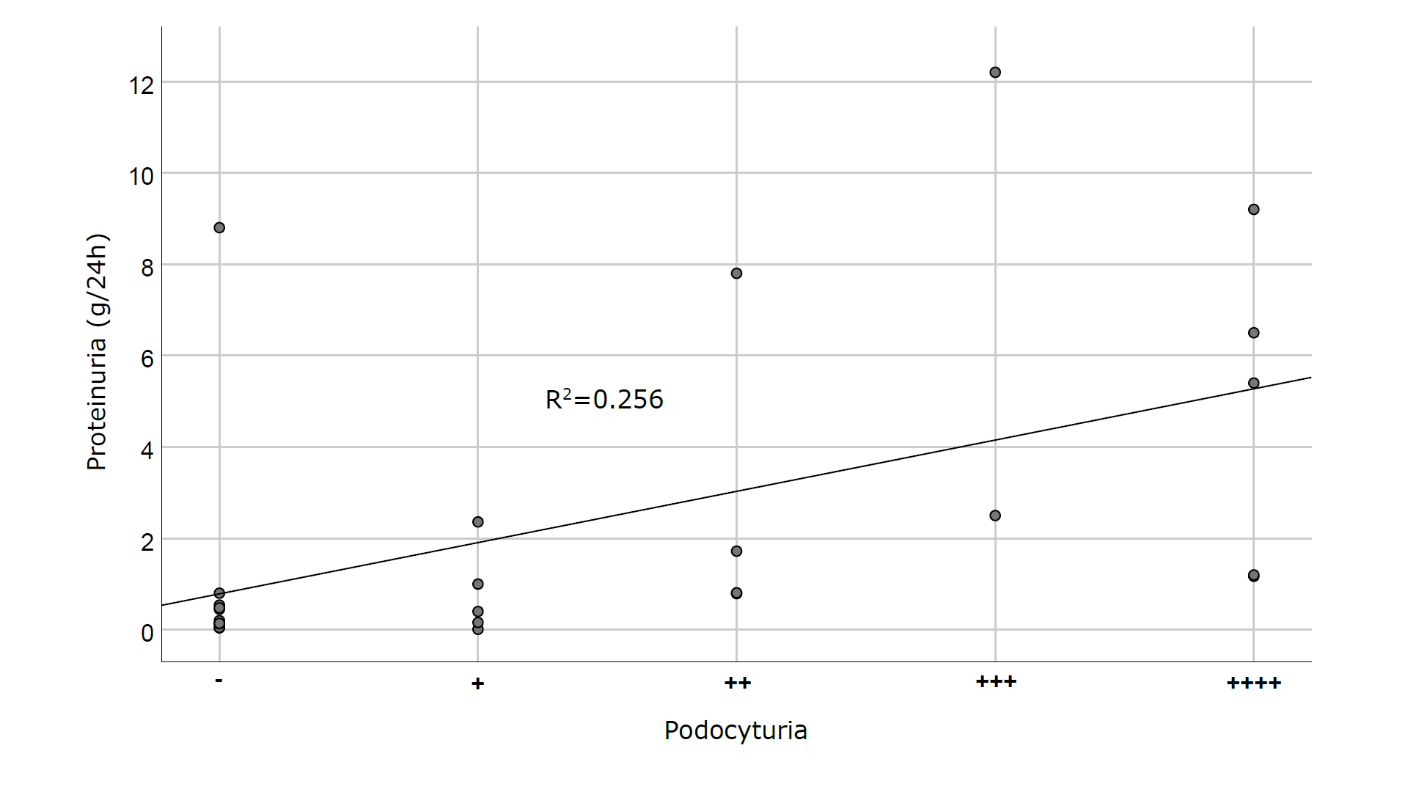


**b)**

**
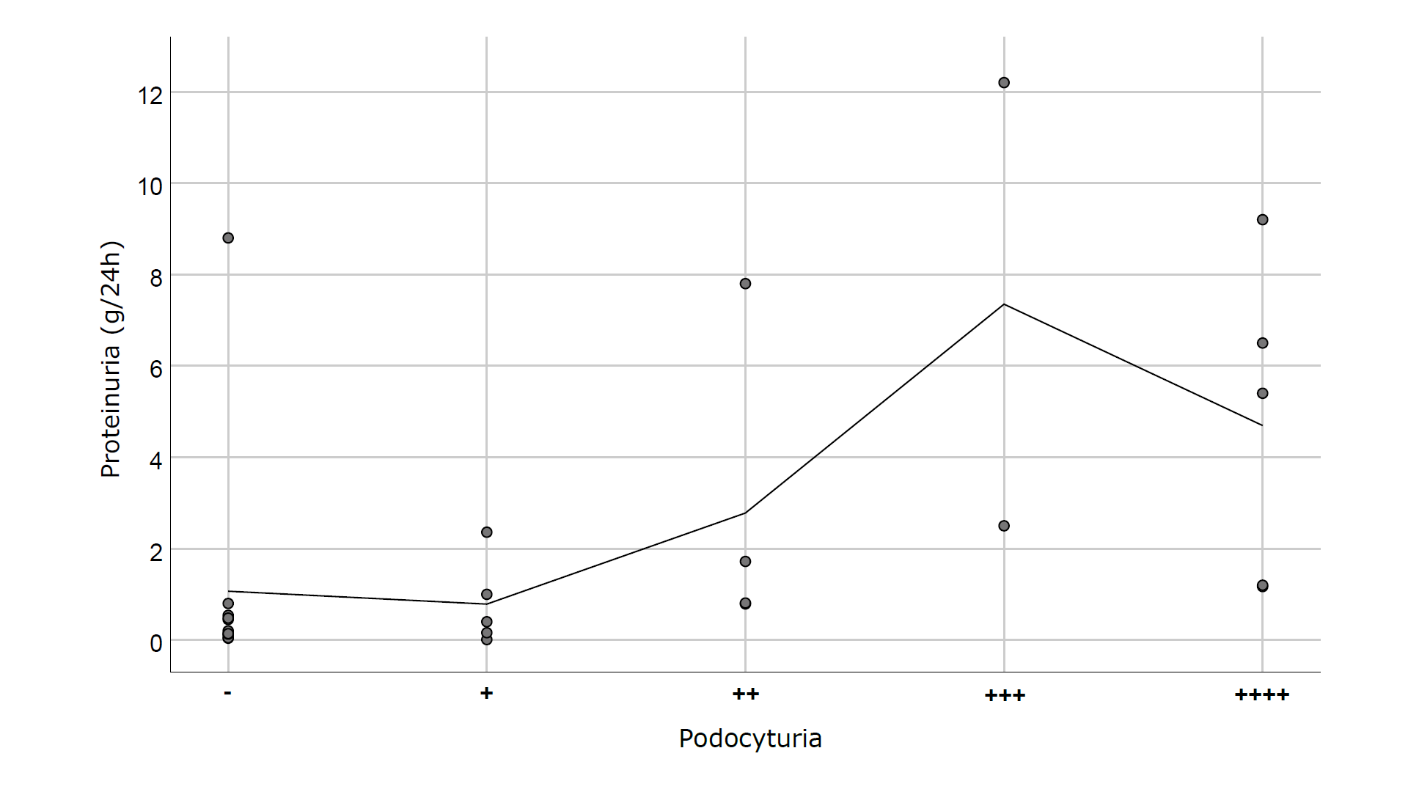
**

**Figure S2**. **Correlation between podocyturia (*continuous values*) and Ab anti-PLA_2_R.** a) As for proteinuria, podocyturia and Ab anti-PLA_2_R seem to not be linearly correlated (R^2^=0.067); b) Schematization of linear trend

**a)**


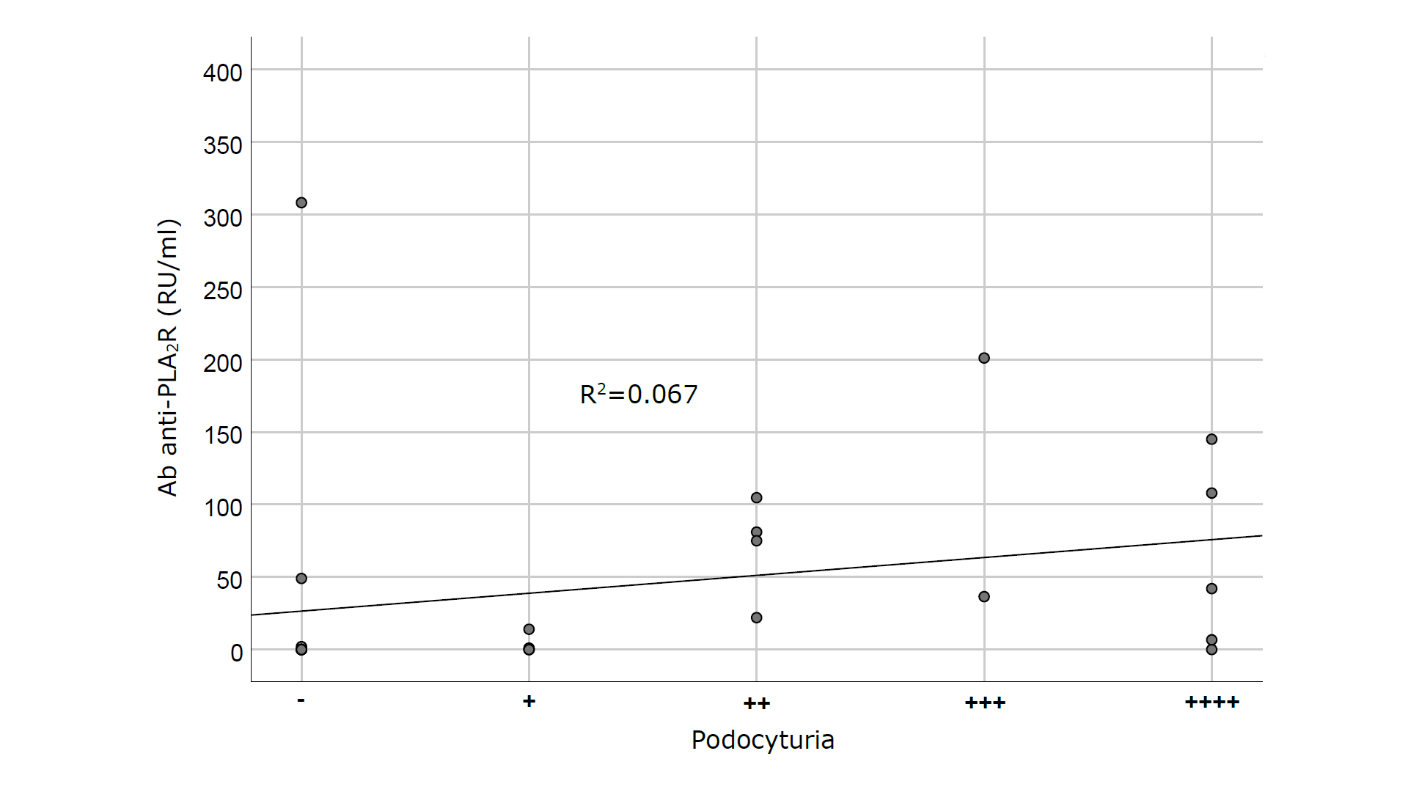


**b)**

**
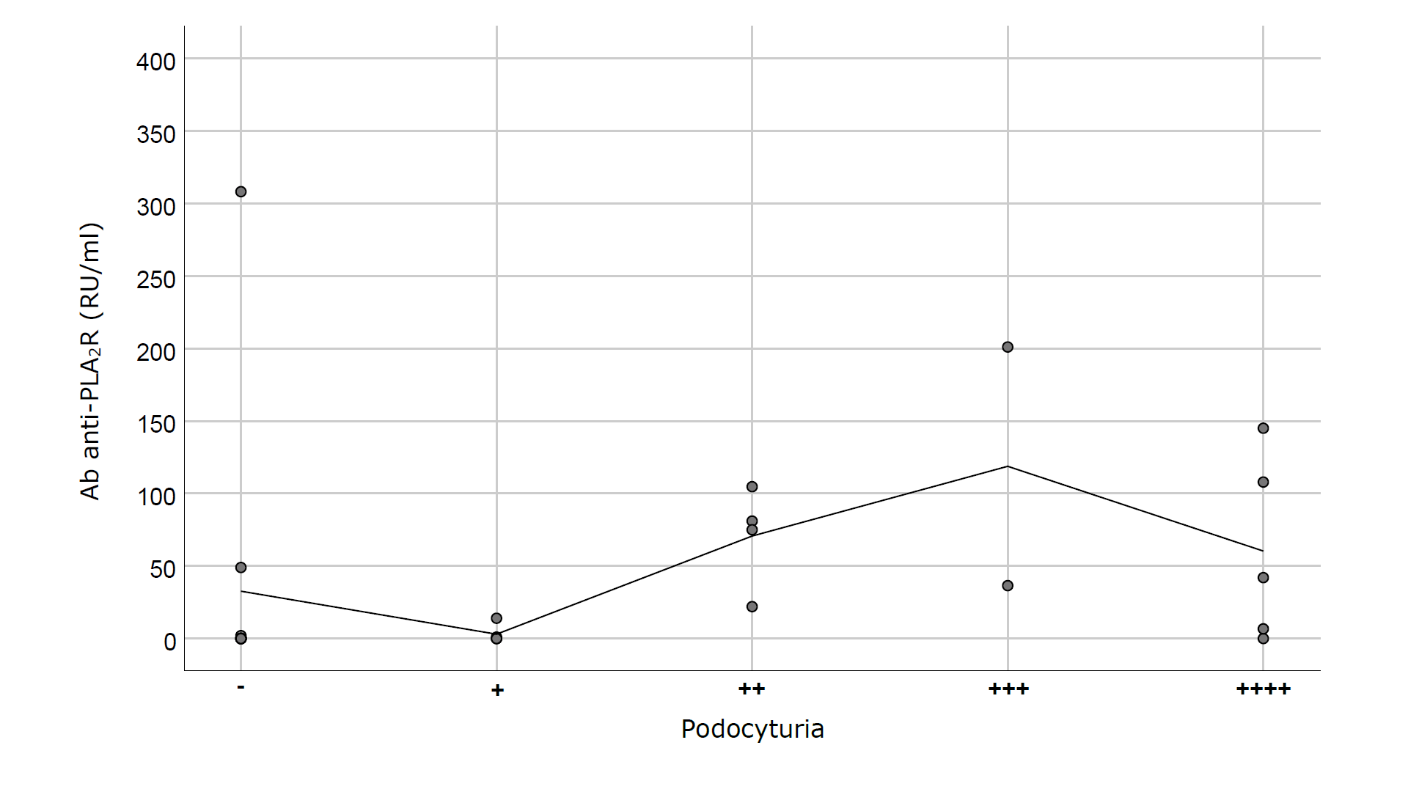
**
